# The Use of Fluorescent Markers to Detect and Delineate Head and Neck Cancer: A Scoping Review

**DOI:** 10.1111/coa.14263

**Published:** 2024-12-04

**Authors:** Akash Srinivasan, Viktorija Kaminskaite, Stuart C. Winter

**Affiliations:** ^1^ Medical Sciences Division University of Oxford Oxford UK; ^2^ Nuffield Department of Surgical Sciences University of Oxford Oxford UK

**Keywords:** clinical trials, fluorescence‐guided surgery, fluorescent marker, head and neck squamous cell carcinoma, intraoperative imaging

## Abstract

**Objectives:**

The aim of surgery for head and neck squamous cell carcinoma (HNSCC) is to achieve clear resection margins, whilst preserving function and cosmesis. Fluorescent markers have demonstrated potential in the intraoperative visualisation and delineation of tumours, such as glioma, with consequent improvements in resection. The purpose of this scoping review was to identify and compare the fluorescent markers that have been used to detect and delineate HNSCC to date.

**Methods:**

A literature search was performed using the Ovid MEDLINE, Ovid Embase, Cochrane CENTRAL, ClinicalTrials.gov and ICTRP databases. Primary human studies published through September 2023 demonstrating the use of fluorescent markers to visualise HNSCC were selected and reviewed independently by two authors.

**Results:**

The search strategy identified 5776 records. Two hundred and forty‐four full texts were reviewed, and sixty‐five eligible reports were included. The most used fluorescent markers in the included studies were indocyanine green (ICG) (*n* = 14), toluidine blue (*n* = 11), antibodies labelled with IRDye800CW (*n* = 10) and 5‐aminolevulinic acid (5‐ALA) (*n* = 8). Toluidine blue and ICG both have limited specificity, although novel targeted options derived from ICG may be more effective. 5‐ALA has been demonstrated as a topical marker and, recently, via enteral administration but it is associated with photosensitivity reactions. The fluorescently labelled antibodies cetuximab‐IRDye800CW and panitumumab‐IRDye800CW are promising options being investigated by ongoing trials.

**Conclusion:**

Multiple safe fluorescent markers have emerged which may aid the surgical resection of HNSCC. Further research in larger cohorts is required to identify which marker should be considered gold standard.

**Summary**

- Fluorescent markers have been utilised in surgical oncology to aid intraoperative visualisation of tumour margins.
- This scoping review identified several fluorescent markers that have been used to detect and delineate head and neck squamous cell carcinoma (HNSCC) to date.
- The most used markers in the included studies were indocyanine green (ICG), toluidine blue, antibodies labelled with IRDye800CW and 5‐aminolevulinic acid (5‐ALA).
- Promising markers that have been researched recently or remain the subject of ongoing trials include 5‐ALA, cetuximab‐IRDye800CW, panitumumab‐IRDye800CW and ONM‐100.
- Further research is required to confirm and compare the safety and efficacy of these markers in larger cohorts.

## 1 Background

Head and neck squamous cell carcinoma (HNSCC) is the sixth most prevalent form of cancer in the world, with a range of severe sequelae including dysphagia, airway obstruction and metastasis [1]. Early‐stage disease can be treated curatively with surgery or radiotherapy [2], whilst advanced disease may require surgery with or without (chemo)radiotherapy [3] and immunotherapy [4, 5]. The oncological and functional outcomes of HNSCC have gradually improved in recent years with more focused treatment of early‐stage disease [6] and a greater emphasis on achieving complete tumour resection with clear margins [7, 8]. However, the surrounding anatomy means that resecting tumours with adequate surgical margins can cause major functional complications, including speech [9] and swallow [10, 11] impairment. Therefore, there is a need for precise intraoperative delineation of tumours to minimise the burden of adjuvant treatment and the risk of recurrence after surgery, whilst preserving quality of life and cosmesis.

Head and neck surgeons primarily rely on palpation, visual assessment and intraoperative frozen section analysis to identify tumour boundaries [12]. However, this approach has limited efficiency and precision, and it may be insufficient for challenging cases such as recurrent tumours [13] and ‘cancers of unknown primary’, in which one or more cancerous lymph nodes are found without a primary mucosal tumour despite extensive investigation [14]. Recently, there has been interest surrounding the use of fluorescent markers to detect and delineate cancer during surgery. The purpose of these markers is to visibly distinguish tumour from healthy tissue to ensure accurate excision margins, thereby reducing the need for adjuvant (chemo)radiotherapy whilst avoiding the functional compromise that a wide resection may cause. A notable example is the use of 5‐aminolevulinic acid (5‐ALA) in neurosurgery to aid the resection of malignant gliomas and improve the rates of overall and progression‐free survival [15, 16]. Fluorescent markers have also demonstrated the potential to identify breast [17], prostate [18] and renal [19] cancer. This has raised questions as to whether fluorescent markers could have a similar role in the visualisation of HNSCC.

There are several settings where fluorescent markers may have utility: during screening to assess the need for biopsies and further investigations, during work‐up to determine the resectability of the tumour, during surgery to delineate tumours and evaluate the excision margins, and during follow‐up to assess for recurrence. The purpose of this review was specifically to identify which fluorescent markers have been clinically evaluated for the intraoperative detection and delineation of HNSCC and summarise the existing evidence regarding their safety and efficacy.

## 2 Methods

A scoping review was performed in accordance with guidance from the Joanna Briggs Institute [20] and reported in line with the Preferred Reporting Items for Systematic Reviews and Meta‐Analyses Extension for Scoping Reviews (PRISMA‐ScR) statement [21]. As this is an emerging topic with a diverse body of literature, a scoping review was considered the most appropriate way to synthesise and summarise the available evidence. Ethical approval was not required. The protocol was registered with the Open Science Framework (https://doi.org/10.17605/OSF.IO/QGVB7).

### 2.1 Search Strategy and Study Selection

Five electronic databases were searched from inception to September 2023: Ovid MEDLINE, Ovid Embase, Cochrane CENTRAL, ClinicalTrials.gov and ICTRP. The search terms are available in Appendix A. Duplicate studies were removed, before two reviewers independently screened the search results using Covidence (Veritas Health Innovation, Melbourne, Australia). Following title and abstract screening, conflicts were resolved by discussion, and this was repeated after full‐text screening.

Table 1 outlines the eligibility criteria for study selection:

**TABLE 1:** Inclusion and exclusion criteria.

Inclusion	Exclusion
Studies demonstrating the use of fluorescent markers to visualise head and neck squamous cell carcinomas.	Studies solely demonstrating the use of fluorescent markers to visualise lymph nodes.
Primary research.	Studies which do not report the administration of a fluorescent marker.
Human in vivo studies.	Studies not available in English.
Trial registrations, protocols, or interim data for ongoing or unpublished clinical trials.	In vitro, ex vivo or non‐human studies.
Conference papers.	Conference abstracts that were later published as full studies.
	Earlier versions of included studies.
	Studies that were withdrawn or terminated.

### 2.2 Data Extraction

Data were independently extracted from each study by two reviewers using a data extraction template. The extracted information included tumour location, the fluorescent marker administered, the route, dose and timing of fluorescent marker administration, the cameras/imaging devices used and the main findings. The in vivo efficacy of the fluorescent markers in detecting HNSCC and the safety data were the primary findings of interest.

### 2.3 Synthesis

As the studies demonstrated significant heterogeneity in terms of methodologies and outcome measures, quantitative analysis was not considered feasible. Consequently, the studies were synthesised qualitatively.

## 3 Results

**FIGURE 1:**
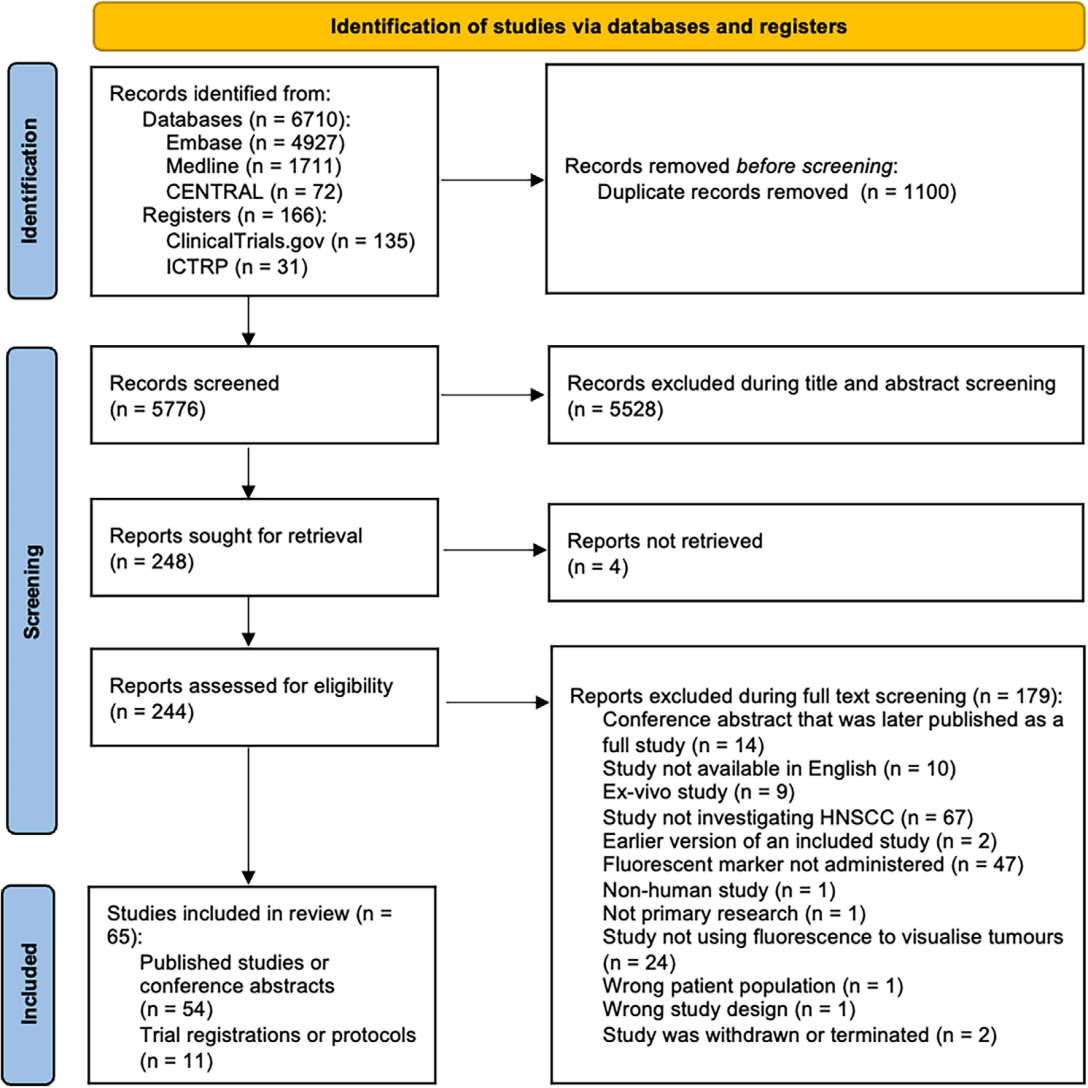
PRISMA flow diagram illustrating the study selection process.

Sixty‐five eligible reports were identified during the study selection process, which is summarised by the PRISMA flow diagram (Figure 1) [22].

The most frequently investigated fluorescent markers in the included publications were indocyanine green (ICG) (*n* = 14) [23, 24, 25, 26, 27, 28, 29, 30, 31, 32, 33, 34, 35, 36], toluidine blue (*n* = 11) [37, 38, 39, 40, 41, 42, 43, 44, 45, 46, 47] and 5‐ALA (*n* = 8) [48, 49, 50, 51, 52, 53, 54, 55]. Recent literature has also studied the use of IRDye800CW [56, 57, 58, 59, 60, 61, 62, 63, 64, 65], by conjugating it with anti‐epidermal growth factor receptor (EGFR) monoclonal antibodies such as cetuximab (*n* = 5) [57, 58, 59, 60, 61] and panitumumab (*n* = 5) [62, 63, 64, 65]. The remaining publications studied Lugol's iodine [66, 67, 68, 69], PARPi‐FL [70], photofrin solution [71, 72], Rose Bengal [73], chlorin E6 [74] and hypericin solution [75]. Trial registrations and protocols involving indocyanine green [76, 77, 78], 5‐ALA [79], Lugol's iodine [80], photofrin solution [81], cetuximab‐IRDye800CW [82], panitumumab‐IRDye800CW [83, 84] and cRGD‐ZW800‐1 [85, 86] were also included in this review.

### 3.1 Toluidine Blue

Toluidine blue is a metachromatic and acidophilic dye, which preferentially stains tissues containing more nucleic acids and wider intracellular canals, such as tumours [87]. Oral rinses containing toluidine blue have been used to aid the detection of oral cavity lesions for decades with minimal adverse effects, as shown by the earliest studies in this review [37, 44, 46]. However, this topical route of administration has generally limited the marker to identifying oral cavity tumours, despite a recent study demonstrating its potential role in detecting glottic cancer [40]. Studies have also highlighted that toluidine blue has a low sensitivity for premalignant disease and submucosal tumour extensions [37, 45, 47], whilst its specificity is limited by a tendency to stain mucin, food particles and purulent exudate [44]. Table 2 summarises the literature evaluating toluidine blue as a diagnostic adjunct for HNSCC.

**TABLE 2:** Summary of the included studies assessing toluidine blue.

Paper	Site of tumours	Fluorescent marker	Imaging system	Timing of intervention	Notable findings	Safety data
Strong et al. [44] Clinical experience narrative	Oral cavity, oropharynx	Toluidine blue Topical 2% solution	N/A	Marker applied followed by examination and biopsy.	Toluidine blue can stain oral and oropharyngeal carcinomas. Specificity is limited by staining of mucin, food particles, purulent exudate and ulcers. Marker cannot delineate deep, submucosal extensions.	N/A
Warnakulasuriya et al. [37] Single‐arm clinical trial	Oral cavity	Toluidine blue Topical 10 mL of 1% solution	N/A	Marker applied as a 20 s rinse followed by examination and photography.	Toluidine blue detected oral squamous cell carcinoma with 100% sensitivity. 5 dysplastic lesions were detected solely using the marker. False‐negative rate for oral premalignant lesions with dysplasia was 20.5%.	N/A
Epstein et al. [46] Single‐arm clinical trial	Oral cavity	Toluidine blue Topical	N/A	Marker applied followed by biopsy.	Toluidine blue was more sensitive than clinical examination at detecting oral carcinoma (100% vs. 78%, *p* = 0.02). No false‐negative results were reported.	N/A
Martin et al. [45] Single‐arm clinical trial	Oral cavity	Toluidine blue Topical	N/A	Marker applied followed by intraoperative evaluation.	Toluidine blue detected invasive malignancy with 100% sensitivity. High‐false negative rates of 42% for carcinoma in situ and 58% for moderate or severe dysplasia, suggesting limited efficacy for premalignant disease.	N/A
Kerawala et al. [47] Single‐arm clinical trial	Oral cavity	Toluidine blue Topical 1% solution	N/A	Marker applied as a 20 s rinse followed by intraoperative evaluation.	All resected margins were free from invasive carcinoma. No patient developed local recurrent disease during the follow‐up period. Marker was unable to identify positive resection margins due to carcinoma in situ or severe dysplasia.	N/A
Onofre et al. [42] Single‐arm clinical trial	Oral cavity	Toluidine blue Topical 1% solution	N/A	Marker applied for 30 s followed by examination and photography.	Toluidine blue detected carcinoma in situ and invasive carcinoma with 100% sensitivity. It demonstrated potential as an adjunct to clinical judgement in choosing biopsy sites.	N/A
Epstein et al. [38] Single‐arm clinical trial	Oral cavity	Toluidine blue Topical 10 mL of 0.5% solution	N/A	Marker applied followed by biopsy.	Toluidine blue increases the sensitivity of detecting carcinoma or carcinoma in situ on biopsy to 96.7% compared to clinical examination alone.	3 patients terminated due to acetic acid intolerance. Reports of discomfort and unpleasant taste.
Epstein et al. [39] Single‐arm clinical trial	Oral cavity	Toluidine blue Topical	N/A	Marker applied followed by biopsy.	After visual examination and chemiluminescence, toluidine blue application reduces the number of false positive biopsies performed without increasing the false‐negative rate.	N/A
Güneri et al. [41] Single‐arm clinical trial	Oral cavity	Toluidine blue Topical 10 mL of 1% solution	N/A	Marker applied as a 20 s rinse with examination and photography before and after.	The addition of toluidine blue and brush cytology to clinical examination increased the detection of carcinoma in situ and squamous cell carcinoma from 62% to 92%.	N/A
Chainani‐Wu et al. [43] Cross‐sectional observational study	Oral cavity	Toluidine blue Topical 1% solution	N/A	Visual examination and VizLite chemiluminescent examination followed by marker application followed by biopsy.	Toluidine blue and clinical assessment for leuko(erythro)plakia lesions may aid in detecting the presence of carcinoma, with a sensitivity of 100% and specificity of 39%.	N/A
Allegra et al. [40] Retrospective study	Glottis	Toluidine blue Topical 1% solution	Standard light surgical microscope, Zeiss	Marker applied for 20 s followed by surgery and histology.	Toluidine blue staining improved the rate of negative resection margins for early glottic cancer treated by transoral laser microsurgery (82.7% in toluidine blue group vs. 52.4% in control group, *p* = 0.047). 5‐year local recurrence‐free survival was 95.6% in the toluidine blue group and 80.9% in the control group (statistical insignificance likely due to the small sample size).	N/A

### 3.2 Indocyanine Green

ICG is a cyanine fluorophore that demonstrates near‐infrared fluorescence [88]. The marker can be safely administered intravenously, with doses ranging up to 5 mg/kg, and it can be visualised using various near‐infrared imaging devices. Alongside its applications in angiography and lymphography, ICG is theorised to accumulate in tumour tissue due to the disrupted vascular endothelium and poorly developed lymphatics in these areas, in a phenomenon known as ‘enhanced permeability and retention’ (EPR) [26, 89]. Overall, there is evidence that the marker does accumulate in tumours of the oral cavity, larynx and pharynx [28, 30, 31, 34], including recurrent tumours [29, 33], but it lacks specificity. To address this limitation, ICG has been conjugated with targeted agents to develop novel markers: ICG‐labelled c‐MET‐binding peptide targets tumours via c‐MET binding [23], whilst ONM‐100/pegsitacianine is activated by the acidic extracellular environment associated with solid tumours [35, 36, 78]. Table 3 summarises the research surrounding the use of ICG to visualise HNSCC.

**TABLE 3:** Summary of the included studies assessing indocyanine green.

Paper	Site of tumours	Fluorescent marker	Imaging system	Timing of intervention	Notable findings	Safety data
Yokoyama et al. [24] Single‐arm clinical trial	Maxillary, oral cavity, oropharynx	Indocyanine green Intravenous 0.5 mg/kg	Near‐infrared camera—HyperEye Medical System, Mizuho Medical Co.	Marker administered followed by fluorescence imaging at 10 mins, 30 mins, 1 h, 2 h, 3 h and 6 h.	Optimum timing for surgery is 30 min to 2 h after ICG injection.	N/A
Chung et al. [30] Single‐arm clinical trial *Conference abstract*	Pharynx, larynx	Indocyanine green Intravenous 7.5 mg	Near‐infrared intraoperative imaging system—SPY/LUNA, Novadaq	Marker administered during surgery. In vivo evaluation prior to resection.	ICG accumulates in head and neck tumours but lacks specificity to serve as a tumour visualisation agent.	N/A
Digonnet et al. [34] Single‐arm clinical trial	Glottis, oropharynx, larynx‐oesophagus, larynx, lip	Indocyanine green Intravenous 0.25 mg/kg	Near‐infrared camera—PDE, Hamamatsu	Marker administered followed by examination after 30–258 min.	ICG and near‐infrared imaging induced primary tumour fluorescence in 4 of the 5 patients.	No adverse events.
Schmidt et al. [25] Single‐arm prospective observational study	Larynx, oropharynx, oral cavity, hypopharynx	Indocyanine green Intravenous 8.3 mg	Near‐infrared endoscopy—IMAGE1 S NIR/ICG system, Karl Storz	Real‐time recording of the procedure, with videos analysed offline.	Near‐infrared ICG endoscopy was feasible for predicting the malignancy of head and neck tumours, with 90.5% sensitivity and 90.9% specificity.	No adverse events.
Von Buchwald et al. [32] Single‐arm prospective clinical trial *Conference abstract*	Oropharynx	Indocyanine green Intravenous	Near‐infrared imaging—Firefly modality incorporated into the Da Vinci Si system	Marker administered followed by intraoperative evaluation.	The experiences with ICG and near‐infrared imaging of oropharyngeal cancer were mixed. The authors reported a potential role for this approach in identifying cancers of unknown primary.	N/A
Scott‐Wittenborn et al. [27] Single‐arm prospective clinical trial	Tongue base, palatine tonsils	Indocyanine green Intravenous 2 × 7.5 mg	Near‐infrared imaging—Da Vinci Firefly system	1st dose of the marker administered followed by immediate visualisation. 2nd dose administered during the resection to identify vascular structures to be avoided.	ICG and the Firefly system did not identify tumour boundaries, unknown primary head and neck cancers, or vascular structures in the oropharynx.	No adverse events.
Stubbs et al. [28] Single‐arm clinical trial	Tongue, tongue base, glossotonsilar sulcus, parotid gland	Indocyanine green Intravenous 5 mg/kg	Near‐infrared imaging—Iridium Camera system, VisionSense	Marker administered followed by surgery after 1 day.	ICG with the VisionSense Iridium system enabled intraoperative visualisation of HNSCC primary tumours, with marked differentiation from normal tissue.	No adverse events.
Cortese et al. [29] Single‐arm clinical trial	Oral cavity, larynx	Indocyanine green Intravenous 0.25 mg/kg	Near‐infrared camera—Artemis, Quest Medical Imaging BV	Marker administered with fluorescence measurements before and 30–45 mins after.	ICG‐based near‐infrared fluorescence mapping was feasible in identifying and delineating tumours in patients previously subjected to radical radiotherapy.	N/A
Pan et al. [31] Single‐arm clinical trial	Tongue, buccal, palate, gingiva, lip, oral cavity	Indocyanine green Intravenous 0.75 mg/kg	Near‐infrared imaging—REAL‐IGS, NuoYuan Medical Devices Co.	Marker administered followed by surgery after 6–8 h.	ICG fluorescence delineated tumour borders from normal tissues in all 20 patients. Unanticipated positive surgical margins were detected in 2 patients.	No adverse events.
Madajewski et al. [36] Phase II single‐arm clinical trial *Conference abstract*	Oral cavity	ONM‐100 (pegsitacianine, polymeric micelles labelled with indocyanine green) Intravenous 0.5–3 mg/kg	Near‐infrared imaging—multiple FDA‐cleared devices	Marker administered followed by surgery on the same day or the day after.	ONM‐100 demonstrated 100% sensitivity and 92% specificity when used during head and neck cancer resections in the oral cavity.	ONM‐100 was well tolerated in a previous Phase 1 clinical trial, but safety data for this Phase 2 study is not provided.
Steinkamp et al. [35] Single‐arm clinical trial	Mandible, oral cavity, tongue, cheek, palate	ONM‐100 (pegsitacianine, polymeric micelles labelled with indocyanine green) Intravenous Multiple dosages as described in a different study	Multiple near‐infrared imaging devices: SPY Elite, Novadaq Explorer Air, SurgVision	Marker administered followed by surgery after 24 ± 8 h.	ONM‐100 achieved clear visual discrimination between tumour and non‐tumour tissue. It demonstrated potential for intraoperative detection of occult disease. The reported sensitivity for tumour‐positive surgical margin detection was 100%.	N/A
De Ravin et al. [26] Case series	Tonsil, tongue base, glossotonsilar sulcus	Indocyanine green Intravenous 5 mg/kg	Near‐infrared imaging—the Da Vinci Xi Firefly endoscope platform was compared with the VisionSense TM Iridium exoscope system	Marker administered followed by surgery after approximately 24 h.	The VisionSense Iridium exoscope system outperformed the Da Vinci endoscope system in visualising ICG‐induced fluorescence.	No adverse events.
Süslü et al. [33] Retrospective study	Hypopharynx	Indocyanine green combined with radiotracer Technetium 99 m Intratumoral 18.5 MBq (in 0.1 mL)	Dual handheld gamma probe and near‐infrared fluorescence imaging system	Marker administered followed by surgery after 2 h.	The combined ICG‐technetium‐99 m tracer aided the removal of a recurrent hypopharyngeal SCC.	The authors believe that the technique is safe, but no data was reported.
Wang et al. [23] Phase I single‐arm clinical trial	Lingual margin, buccal, gum, maxilla	c‐MET‐binding peptide‐indocyanine green Topical 25 mL of 2.5 μM or 5 μM solution	Endoscopic camera and an ICG‐optimised LED filter system	Marker administered followed by surgery the day after. Videos acquired before application, after application before a cleansing wash, and after a cleansing wash.	Topical cMET‐binding peptide‐ICG is feasible for visualising oral squamous cell carcinoma and detecting margins. The 5 μM concentration was associated with better results.	No grade I or higher adverse events.
Thamboo et al. [76] Prospective case series and feasibility study *Trial registration, not yet recruiting*	Sinus	Indocyanine green Intravenous, intralesional 1.25 mg, 2.5 mg, 3.75 mg, 5 mg, 6.25 mg (at discretion of surgeon), 7.5 mg (at discretion of surgeon)	N/A	Intraoperative margin estimation with the naked eye followed by marker administration.	N/A	Adverse events will be recorded for the secondary outcome.
Sumer et al. [78] Phase IIa single‐dose open‐label clinical trial *Trial registration, not yet recruiting*	N/A *Part 2 of the study will investigate patients with cancer of unknown primary*.	ONM‐100 (pegsitacianine, polymeric micelles labelled with indocyanine green) 1 mg/kg	Laryngoscopy and panendoscopy with near‐infrared cameras	Marker to be administered followed by surgery after 6–100 h.	N/A	Patient safety will be assessed for 10 days (± 48 h) post‐dose.
Roussy et al. [77] Prospective non‐randomised bicentric study *Trial registration, unknown status*	Oral cavity, oropharynx	Indocyanine green 25 mg/10 mL	Near‐infrared imaging	Imaging to be performed real‐time to assess for residual disease after resection.	N/A	N/A

### 3.3 5‐Aminolevulinic Acid

5‐ALA is an amino acid that is intracellularly metabolised via the haem‐synthesis pathway to form protoporphyrin IX, a by‐product that exhibits violet‐red fluorescence after excitation with blue light [88]. The relatively high metabolism of tumour cells means that they produce and accumulate larger quantities of this fluorophore after 5‐ALA administration compared with normal tissue [15]. The use of 5‐ALA to detect HNSCC has been demonstrated for over two decades, with the earliest formulations ranging from topical gels [49] to nebulised solutions [54, 55]. More recently, enteral 5‐ALA was shown to induce strong intraoperative fluorescence of HNSCC and reveal positive margins and perineural invasion [53]. However, the photosensitising action of 5‐ALA can cause adverse reactions, such as the erythematous and desquamating rashes reported following enteral administration [53]. Table 4 summarises the studies investigating whether 5‐ALA can aid the visualisation of HNSCC.

**TABLE 4:** Summary of the included studies assessing 5‐aminolevulinic acid.

Paper	Site of tumours	Fluorescent marker	Imaging system	Timing of intervention	Notable findings	Safety data
Leunig et al. [48] Single‐arm clinical trial	Oral cavity	5‐aminolevulinic acid Topical 200 mg (0.4% solution)	Fluorescence endoscopy—Optronics VI 470, Karl Storz	Marker applied followed by fluorescence measurements between 0 and 3 h.	5‐ALA‐induced fluorescence in all patients. Maximum fluorescence was observed after 1–2 h. The tumour‐to‐healthy tissue fluorescence intensity ratio was consistently 10:1.	N/A
Mehlmann et al. [55] Single‐arm clinical trial	Larynx	5‐aminolevulinic acid Topical (inhaled via nebuliser) Solution of 30 mg 5‐ALA dissolved in 5 mL 0.9% NaCl	Optimised endoscope—D‐Light‐AF System, Karl Storz	Marker administered followed by fluorescence imaging after 1–2 h.	Topical 5‐ALA‐induced fluorescence in laryngeal cancers with 95% sensitivity and 80% specificity. The inhalational route avoids the previously described time‐consuming topical application of markers under general anaesthesia.	The local application of 5‐ALA and rapid metabolism of induced protoporphyrin IX minimised unwanted photosensitivity.
Csanády et al. [52] Single‐arm clinical trial	Larynx, pharynx	5‐aminolevulinic acid Topical 1% solution	Direct fluorescence endoscopy and laryngomicroscopy—D Light System, Karl Storz	Marker applied followed by fluorescence imaging after 1.5–2 h.	5‐ALA fluorescence imaging demonstrated 96% sensitivity and 76% specificity for laryngeal and pharyngeal tumours.	No adverse events.
Zheng et al. [51] Single‐arm clinical trial	Oral cavity	5‐aminolevulinic acid Topical 0.4% solution	Digitised fluorescence endoscopic imaging system—D Light AF System, Karl Storz	Marker applied as a 15‐min rinse followed by fluorescence imaging after 1.5–2 h.	5‐ALA fluorescence imaging and quantifying the images with the red‐to‐blue intensity ratio enables benign oral lesions and different stages of malignancy to be accurately differentiated.	No adverse events.
Arens et al. [54] Single‐arm two‐step prospective clinical trial	Larynx	5‐aminolevulinic acid Topical (inhaled) 0.6% solution	70° rigid‐angled endoscope with an integrated filter and the D‐light‐AF System, Karl Storz	Marker administered followed by fluorescence imaging during laryngoscopy.	5‐ALA fluorescence endoscopy was slightly more sensitive than autofluorescence endoscopy (97% vs. 94%). Marker was also better at identifying recurrent cancer lesions in scarred vocal folds. Specificity may be limited by greater accumulation of protoporphyrin IX in inflamed tissues.	The authors report that cutaneous sensitisation can be reduced or avoided by local application of 5‐ALA.
Morawiec‐Sztandera et al. [50] Double‐arm clinical trial *Conference abstract*	Oral cavity, pharynx, larynx	5‐aminolevulinic acid Topical	N/A	N/A	5‐ALA had 76% sensitivity and 86% specificity in diagnosing neoplastic tumours of the oral cavity, pharynx and larynx.	N/A
Sadykov et al. [49] Single‐arm clinical trial *Conference abstract*	Oropharynx	5‐aminolevulinic acid Topical 20% gel	Illumination of λ = 405 +/− 5 nm light	Marker administered followed by evaluation after 3 h.	5‐ALA aids the early detection of primary and recurrent oropharyngeal tumours, with 53% sensitivity and 80% specificity.	N/A
Filip et al. [53] Single‐arm prospective pilot trial	Nasal cavity, oral cavity, subglottis	5‐aminolevulinic acid Oral/enteral 20 mg/kg dissolved in 100 mL of sterile water	405 nm blue light fluorescence‐guided headlight system and the operating microscope with blue light capabilities	Marker administered followed by induction of anaesthesia after 3–5 h and intraoperative evaluation.	Enteral 5‐ALA induced robust intraoperative fluorescence of HNSCC in 6 out of 7 patients. Marker detects positive margins, perineural invasion and metastatic nodal disease.	Acute erythematous, desquamating rashes in two patients and mild liver function test elevation in one patient. No severe or long‐term reactions.
Miyamoto et al. [79] Single‐arm clinical trial *Trial registration, completed*	N/A	5‐aminolevulinic acid Oral/enteral 20 mg/kg	N/A	Marker to be administered pre‐operatively.	N/A	Complications to be recorded as the secondary outcome.

### 3.4 Fluorescent Antibodies

IRDye800CW is another cyanine fluorophore displaying near‐infrared fluorescence, but it has a higher solubility and fluorescence intensity than ICG [88]. Moreover, IRDye800CW can be conjugated with monoclonal antibodies to target molecules that are highly expressed by HNSCC cells, such as EGFR [90]. The most notable examples of fluorescently labelled anti‐EGFR antibodies are cetuximab‐IRDye800CW and panitumumab‐IRDye800CW. These markers are intravenous formulations and a variety of doses have been tested in dose‐escalation trials [57, 59, 60, 65], although lower doses are sufficient when the fluorescence imaging system is correctly optimised for IRDye800CW detection. Both markers are capable of inducing HNSCC fluorescence and differentiating tumour tissue from normal tissue, with reported examples of panitumumab‐IRDye800CW improving surgical decision‐making and enabling the resection of unanticipated tumour tissue [64]. It has been suggested that panitumumab‐IRDye800CW is safer than cetuximab‐IRDye800CW [65], but both markers remain under investigation by ongoing trials [82, 83, 84]. Table 5 summarises the studies assessing the use of these markers to detect HNSCC.

**TABLE 5:** Summary of the included studies assessing fluorescently labelled antibodies.

Paper	Site of tumours	Fluorescent marker	Imaging system	Timing of intervention	Notable findings	Safety data
Rosenthal et al. [59] Phase I single‐arm clinical trial	Oral cavity, nasal cavity, oropharynx, lip	Cetuximab‐IRDye800CW Intravenous 2.5 mg/m^2^, 25 mg/m^2^, or 62.5 mg/m^2^	Wide‐field optical imaging device designed for ICG imaging—LUNA Imaging System, Novadaq	Marker administered followed by imaging on day 0 and day 1 in clinic and intraoperatively.	Cetuximab‐IRDye800CW differentiated tumour from normal tissue during resection. Average tumour‐to‐background fluorescence ratio was 5.2 in the highest dose range.	Grade I adverse events: elevated AST, tumour redness, tumour swelling, sinus bradycardia, dizziness, ECG changes, tumour pain, hypomagnesaemia, tumour burning and hypotension. No grade II adverse events.
Moore et al. [60] Single‐arm dose‐escalating clinical trial	Oral cavity	Cetuximab‐IRDye800CW Intravenous 2.5 mg/m^2^, 25 mg/m^2^, or 62.5 mg/m^2^	Wide‐field optical imaging device designed for ICG imaging—LUNA Imaging System, Novadaq	Marker administered followed by imaging after 3 h and every 24 h thereafter in clinic, and on the day of surgical resection (day 3–7).	Cetuximab‐IRDye800CW facilitated pre‐operative mapping of tumour borders and occult lesion detection. Fluorescence contrast between tumour and normal tissue was highest 1 day after marker administration.	N/A
Moore et al. [61] Single‐arm clinical trial	Oral cavity	Cetuximab‐IRDye800CW Intravenous 25 mg/m^2^ *This was preceded by either 10 mg or 100 mg unlabelled cetuximab*.	Near‐infrared open‐field imaging—Novadaq LUNA imaging system	Marker administered as a 1‐h infusion followed by surgery after 3–4 days.	Administering a preload of unlabelled cetuximab enhanced the effectiveness of fluorescently labelled cetuximab. 100 mg unlabelled cetuximab was superior to 10 mg.	One patient in each group experienced grade I adverse events. 10 mg group: dizziness, ECG changes, tumour pain, hypomagnesaemia. 100 mg group: ECG changes, elevated AST, hypomagnesaemia.
Gao et al. [65] Phase I single‐centre non‐randomised prospective study	Oral cavity	Panitumumab‐IRDye800CW Intravenous 0.06 mg/kg, 0.5 mg/kg, 1 mg/kg, or 50 mg	Two wide‐field optical imaging systems modified for IRDye800 fluorescence imaging: PINPOINT, Novadaq Explorer Air, SurgVision	Marker administered followed by surgery after 1–5 days.	Panitumumab‐IRDye800CW had > 95% sensitivity and > 98% NPV for detecting areas where the tumour tissue was ≤ 1 mm from the margin edge.	One grade I adverse event in the 0.06 mg/kg cohort: QT prolongation after drug infusion which returned to baseline at the 30‐day follow‐up. The authors say that these results show improved safety over cetuximab‐IRDye800CW.
Van Keulen et al. [64] Phase I single‐arm clinical trial	Lateral tongue, retromolar trigone, buccal, hard palate, floor of the mouth, maxillary sinus, scalp	Panitumumab‐IRDye800CW Intravenous	Handheld near‐infrared fluorescence imaging device—Novadaq	Marker administered followed by surgery after 1–5 days.	Panitumumab‐IRDye800CW improved surgical decision‐making in 3 cases (21.4%) by visualising unanticipated tumour tissue.	N/A
Moore et al. [56] Single‐arm clinical trial *Conference abstract*	N/A	Anti‐EGFR antibodies conjugated to IRDye800CW	Da Vinci Xi robot with integrated near‐infrared fluorescence imaging technology	Marker administered followed by surgery.	Cancer‐specific fluorescence was observed with tumour‐to‐background ratios > 2.0 in all cases except 1. Markers identified residual cancer in 2 patients, enabling further resection.	N/A
Voskuil et al. [57] Phase I single‐arm clinical trial	Buccal, floor of the mouth, tongue	Cetuximab‐IRDye800CW Intravenous Single‐dose cohorts (10 mg, 25 mg, or 50 mg of marker). Pre‐dosed cohorts (75 mg unlabelled cetuximab +15 mg or 25 mg of marker after 1 h).	Custom‐built fluorescence endoscopy platform attached to a flexible nasendoscope—SurgVision Intraoperative fluorescence camera system—Explorer Air, SurgVision	Marker administered followed by surgery after 4 days.	75 mg unlabelled cetuximab followed by 15 mg cetuximab‐IRDye800CW was optimal based on safety and fluorescence. The use of equipment optimised for IRDye800CW detection allowed for lower doses than previously reported.	5 grade I adverse events were reported. *The 1 grade II event was considered iatrogenic rather than imaging agent‐related, as administration speed was accidentally set too fast*.
Rao et al. [63] Single‐arm clinical trial *Conference abstract*	Oropharynx	Panitumumab‐IRDye800CW Intravenous	Near‐infrared technology—Da Vinci Xi robot camera	Marker administered followed by surgery after 48 h.	Panitunumab‐IRDye800CW can detect oropharyngeal squamous cell carcinoma even when EGFR expression is low.	N/A
Stone et al. [63] Single‐arm clinical trial *Conference abstract*	Oropharynx	Panitumumab‐IRDye800CW Intravenous 50 mg	Near‐infrared technology—Da Vinci Xi robot camera	Marker administered followed by surgery after 48 h.	Panitimumab‐IRDye800CW combined with fluorescence quantification can improve the accuracy and precision of tumour resection.	N/A
Zhou et al. [62] Phase I/II open‐label single‐arm clinical trials	N/A	Panitumumab‐IRDye800CW Intravenous 50 mg	Near‐infrared imaging—SPY fluorescence imaging platform, Novadaq	Marker administered followed by surgery after 1–3 days.	Near‐infrared imaging enhanced HNSCC tumour contrast relative to white‐light illumination by 3.4‐fold (*p* < 0.0001).	Adverse events were collected up to 30 days after infusion, but none are reported in this paper.
De Wit et al. [58] Phase II single‐arm clinical trial	Tongue, mandibular gingiva, maxillary gingiva, floor of mouth, cheek, buccal fold, glossotonsilar sulcus	Cetuximab‐IRDye800CW Intravenous 15 mg *This was preceded by 2 mg clemastine and 75 mg unlabelled cetuximab an hour earlier*.	Explorer Air, SurgVision	Marker administered followed by surgery after 2 days.	Cetuximab‐IRDye800CW caused all tumours to show increased fluorescence signal compared to adjacent normal tissue, with a median signal‐to‐background ratio of 3.1.	Four adverse events (5%) during administration of the pre‐dose cetuximab, including two serious events (anaphylactic reaction with hypotension) and one grade I event (rash, minimal angioedema). The other grade I event was unrelated to the study drugs.
Thomas et al. [83] Phase II single‐arm clinical trial *Trial registration, recruiting*	N/A	Panitumumab‐IRDye800CW Intravenous 50 mg	N/A	Marker to be infused over 60 min. Fluorescence to be measured between day 0 and day 15.	N/A	N/A
Rosenthal et al. [84] Phase I single‐arm clinical trial *Trial registration, recruiting*	N/A	Panitumumab‐IRDye800CW Intravenous *A radioactive marker called indium In 111 panitumumab will also be used for the second imaging modality of SPECT/CT*.	Fluorescence imaging, in combination with SPECT/CT	Fluorescent marker to be administered before the radioactive marker on day 0. SPECT/CT to be performed between days 1–5 followed by standard of care surgery with fluorescence imaging.	N/A	The number of grade II or higher adverse events that are definitely or probably related to the study drugs will be recorded.
Witjes et al. [82] Phase II single‐arm clinical trial *Trial registration, not yet recruiting*	Oral cavity	Cetuximab‐IRDye800CW 15 mg *This will be preceded by 75 mg unlabelled cetuximab*.	N/A	Imaging will be performed in real‐time intraoperatively.	N/A	N/A

### 3.5 Other Fluorescent Markers

A novel marker that is generating interest is cRGD‐ZW800‐1. ZW800‐1 itself is a zwitterionic fluorophore that demonstrates near‐infrared fluorescence, whilst cRGD is a cyclic pentapeptide which binds to specific tumour cell integrins and tumour‐associated vascular endothelium [91]. A 2020 study combined ZW800‐1 with cRGD to form cRGD‐ZW800‐1 and successfully demonstrated its ability to intraoperatively visualise colon cancer. There are two ongoing phase II clinical trials studying the use of cRGD‐ZW800‐1 to guide surgery for oral cancer [85], as well as laryngeal and hypopharyngeal cancer [86].

Lugol's iodine reacts with cytoplasmic glycogen to produce a colour change in normal tissue, but the increased keratinisation and reduced glycogen content of cancer cells prevents staining [92]. This marker can aid the detection of malignant oral tumours [66, 67] and reduce the likelihood of unsatisfactory surgical margins [68, 69], but the effect on recurrence and survival rates is unclear [66, 67]. Moreover, iodine has irritant effects and a case of marked oropharyngeal ulceration has been reported [68]. An alternative topical marker is PARPi‐FL, which highlights the overexpression of poly‐ADP ribose polymerase 1 (PARP1) in tumours [70]. This fluorophore appears to be well tolerated, and it is the subject of an ongoing phase II trial [70]. The remaining markers identified by this review are not currently being investigated in trials and appear unlikely to be favoured options in the future.

## 4 Discussion

The head and neck cancer surgeon must skilfully balance the removal of all cancerous tissue with clear resection margins alongside the need to preserve form and function [7, 8]. A distance of greater than 5 mm between the invasive carcinoma and the surgical margins on histology is defined as clear by the Royal College of Pathologists, whereas 1–5 mm is a close margin and less than 1 mm is an involved margin [93]. Involved and close margins have significant prognostic implications, including a higher risk of locoregional recurrence and reduced overall survival [94]. The management is also impacted, as re‐resection and/or adjuvant (chemo)radiotherapy is typically required which carries a further risk of functional complications [94]. Although surgeons have managed to achieve a degree of success through palpation and visual assessment alone, involved resection margins remain a frequent occurrence with reported rates as high as 23% [93, 95], and the oropharynx, hypopharynx and larynx are particularly high‐risk locations [95]. A potential solution is the use of fluorescent markers to visualise cancer tissue and provide valuable intraoperative guidance. Viable markers should selectively target the cancer, accumulate sufficiently within the tissue to generate fluorescence, and distinguish it from normal tissue. The findings of this scoping review reveal several fluorescent markers that display these properties in the detection of HNSCC.

The most studied fluorescent markers to date are toluidine blue, ICG, 5‐ALA and IRDye800CW conjugated with anti‐EGFR antibodies. Toluidine blue was highlighted by the earliest papers in this review, whereas it has only been studied once in the last five years [40] and there are limitations regarding its specificity and its ability to detect premalignant disease. ICG is a safe, FDA‐ and EMA‐approved marker with several clinical applications [96], but its use to detect HNSCC has generated mixed results and there are concerns over its specificity [27, 30, 32]. However, novel targeted markers derived from ICG including ONM‐100 and cMBP‐ICG may be more effective for specific tumour visualisation. 5‐ALA is another safe marker which is licensed by the FDA and EMA for glioma surgery [97], and the recent experiences of Filip et al. [53] suggest that it may have a role in HNSCC surgery too. Finally, the emergence of fluorescently labelled cetuximab and panitumumab has generated recent excitement, with multiple ongoing clinical trials [82, 83, 84]. There remains a lack of research directly comparing these fluorescently labelled antibodies, although panitumumab‐IRDye800CW may be safer [65] and notably demonstrated the ability to improve surgical decision‐making [64]. This has led Thomas et al. [83] to describe panitumumab‐IRDye800CW as the ‘frontrunner in optical imaging’.

The markers described in this review are administered in different ways, which create various benefits and challenges. Topical toluidine blue has minimal associated adverse effects, yet this route of administration limits its ability to identify tumours outside the oral cavity and extensions of tumours beneath the surface [44]. 5‐ALA was initially administered as a topical or nebulised solution, but the enteral route recently showed promise despite the more significant adverse reactions [53]. Meanwhile, ICG, cetuximab‐IRDye800CW and panitumumab‐IRDye800CW are delivered intravenously and this can occasionally result in significant systemic reactions [58]. Nevertheless, the systemic route may be necessary to aid the assessment of deep margins [31, 35, 64] and for identifying cancers of unknown primary [32, 78].

Alongside the fluorescent marker, a compatible imaging system is often required to visualise the tumour. Several near‐infrared imaging systems have been exhibited in the studies assessing ICG and the fluorescently labelled antibodies, whereas 5‐ALA fluorescence is typically visualised using blue light imaging. The choice of imaging system also depends on tumour location [57] and whether in vivo or ex vivo imaging is being performed [35]. Developing new imaging systems is a challenge which limits the speed at which new markers can be trialled and approved. To overcome this, there are examples where existing systems designed for ICG were repurposed for IRDye800CW imaging [59, 60]. However, the findings of Voskuil et al. [57] highlight the importance of compatible imaging devices to allow for lower doses of the marker and achieve better outcomes.

This scoping review has limitations. Firstly, the manual nature of the systematic search and screening process may have resulted in mistakenly excluded studies. Secondly, the statuses of the included ongoing clinical trials may have changed or updated since the point when data extraction was performed. Finally, this review used broad inclusion criteria to capture a range of information, but the heterogeneous nature of the included studies did not allow for quantitative analysis. This provides a future opportunity for focused systematic reviews on this topic.

## 5 Conclusion

Numerous fluorescent markers have emerged which may aid the detection and delineation of HNSCC and facilitate improvements in surgical resection. Presently, the most promising options include 5‐ALA, cetuximab‐IRDye800CW, panitumumab‐IRDye800CW and targeted ICG derivatives such as ONM‐100. Randomised controlled trials in larger cohorts are required to determine whether these markers improve outcomes compared to standard of care and identify which marker should be the gold standard for visualising HNSCC.

## Author Contributions

A.S., V.K. and S.C.W. designed the work; A.S. and V.K. acquired and analysed data; AS drafted the manuscript; A.S., V.K. and S.C.W. revised, and approved the manuscript; A.S., V.K. and S.C.W. agree to be accountable for all aspects of the work.

## Ethical Statement

This study is a scoping review of previously published articles. No patient‐identifiable data were included, and no ethical approval was required.

## Conflicts of Interest

The authors declare no conflicts of interest.

### Peer Review

The peer review history for this article is available at https://www.webofscience.com/api/gateway/wos/peer‐review/10.1111/coa.14263.

## Supporting information


Data S1.

## Data Availability

Data sharing not applicable to this article as no datasets were generated or analysed during the current study.
