# Supplementary material for: The Use of Fluorescent Markers to Detect and Delineate Head and Neck Cancer: A Scoping Review
Source: Clin Otolaryngol. 2024 Dec 4;50(2):220–40. doi: 10.1111/coa.14263 (PMC11792437; doi:10.1111/coa.14263)
Supplement: Supplementary file 1 — Data S1. [file COA-50-220-s001.docx]

**The use of fluorescent markers to detect and delineate head and neck cancer: a scoping review**

**Appendix A:** **full search strategy and results of the literature search**

**Ovid MEDLINE**

| **Lines** | **Search term** | **Search results** |
| --- | --- | --- |
| 1 | coloring agents/ or exp fluorescent dyes/ or exp photosensitizing agents/ | 212777 |
| 2 | (colo?ring agent* or fluoresc* dye or fluoresc* dyes or photosensiti?ing agent* or photo-sensiti?ing agent* or photosensit?er* or fluoresc* stain* or fluoresc* label* or (fluoresc* adj4 biomarker*) or (fluoresc* adj4 bio-marker*) or (fluoresc* adj4 marker*)).mp. | 202731 |
| 3 | aminolevulinic acid/ or Indocyanine Green/ | 16396 |
| 4 | (aminolevulinic acid or 5-aminolevulinic acid or 5-ALA or indocyanine green or ICG).mp. | 28516 |
| 5 | Optical Imaging/ | 15080 |
| 6 | (optical imaging or fluoresc* imaging or fluorescence-guided surger* or (intraoperative* adj4 fluoresc*) or (intra-operative* adj4 fluoresc*)).mp. | 40776 |
| 7 | 1 or 2 or 3 or 4 or 5 or 6 | 300716 |
| 8 | "head and neck neoplasms"/ or "squamous cell carcinoma of head and neck"/ or otorhinolaryngologic neoplasms/ or laryngeal neoplasms/ or exp pharyngeal neoplasms/ | 127453 |
| 9 | exp Neoplasms/ | 3875144 |
| 10 | Mouth Mucosa/ or exp Pharynx/ or exp Larynx/ or Otolaryngology/ | 132889 |
| 11 | (otorhinolaryngolog* or otolaryngolog* or head or heads or neck* or tonsil* or throat* or nasal cavit* or laryn* or glotti* or vocal cord* or pharyn*).mp. | 863382 |
| 12 | 9 and (10 or 11) | 198121 |
| 13 | ((otorhinolaryngolog* or otolaryngolog* or head or heads or neck* or tonsil* or throat* or nasal cavit* or laryn* or glotti* or vocal cord* or pharyn*) adj4 (cancer* or tumo?r* or neoplasm* or carcinoma* or malignan*)).mp. | 142222 |
| 14 | (HNSCC or SCCHN).mp. | 13308 |
| 15 | 8 or 12 or 13 or 14 | 233628 |
| 16 | 7 and 15 | 1854 |
| 17 | 16 not (exp animals/ not humans/) | 1711 |

**Ovid Embase**

| **Lines** | **Search term** | **Search results** |
| --- | --- | --- |
| 1 | exp coloring agent/ or exp fluorescent dye/ or exp photosensitizing agent/ | 418307 |
| 2 | (colo?ring agent* or fluoresc* dye or fluoresc* dyes or photosensiti?ing agent* or photo-sensiti?ing agent* or photosensit?er* or fluoresc* stain* or fluoresc* label* or (fluoresc* adj4 biomarker*) or (fluoresc* adj4 bio-marker*) or (fluoresc* adj4 marker*) or aminolevulinic acid or 5-aminolevulinic acid or 5-ALA or indocyanine green or ICG).mp. | 198692 |
| 3 | fluorescence imaging/ | 36533 |
| 4 | (optical imaging or fluoresc* imaging or fluorescence-guided surger* or (intraoperative* adj4 fluoresc*) or (intra-operative* adj4 fluoresc*)).mp. | 57678 |
| 5 | 1 or 2 or 3 or 4 | 512303 |
| 6 | "head and neck tumor"/ or "head and neck cancer"/ or exp larynx tumor/ or exp pharynx tumor/ | 151983 |
| 7 | exp neoplasm/ | 5531646 |
| 8 | mouth mucosa/ or exp pharynx/ or exp larynx/ or otorhinolaryngology/ | 163416 |
| 9 | (otorhinolaryngolog* or otolaryngolog* or head or heads or neck* or tonsil* or throat* or nasal cavit* or laryn* or glotti* or vocal cord* or pharyn*).mp. | 1199278 |
| 10 | 7 and (8 or 9) | 336832 |
| 11 | ((otorhinolaryngolog* or otolaryngolog* or head or heads or neck* or tonsil* or throat* or nasal cavit* or laryn* or glotti* or vocal cord* or pharyn*) adj4 (cancer* or tumo?r* or neoplasm* or carcinoma* or malignan*)).mp. | 187063 |
| 12 | (HNSCC or SCCHN).mp. | 20350 |
| 13 | 6 or 10 or 11 or 12 | 374320 |
| 14 | 5 and 13 | 5398 |
| 15 | 14 not ((exp animal/ or nonhuman/) not exp human/) | 4927 |

**Cochrane CENTRAL**

| **Lines** | **Search term** | **Search results** |
| --- | --- | --- |
| 1 | MeSH descriptor: [Coloring Agents] this term only | 759 |
| 2 | MeSH descriptor: [Fluorescent Dyes] explode all trees | 207 |
| 3 | MeSH descriptor: [Photosensitizing Agents] explode all trees | 1006 |
| 4 | ((colo?ring NEXT agent*) or (fluoresc* NEXT dye) or (fluoresc* NEXT dyes) or (photosensiti?ing NEXT agent*) or (photo-sensiti?ing NEXT agent*) or photosensit?er* or (fluoresc* NEXT stain*) or (fluoresc* NEXT label*) or (fluoresc* NEAR/3 biomarker*) or (fluoresc* NEAR/3 bio-marker*) or (fluoresc* NEAR/3 marker*)):ti,ab,kw | 3160 |
| 5 | MeSH descriptor: [Aminolevulinic Acid] this term only | 564 |
| 6 | MeSH descriptor: [Indocyanine Green] this term only | 317 |
| 7 | ("aminolevulinic acid" or "5-aminolevulinic acid" or "5-ALA" or "indocyanine green" or "ICG"):ti,ab,kw | 2499 |
| 8 | MeSH descriptor: [Optical Imaging] this term only | 100 |
| 9 | ("optical imaging" or (fluoresc* NEXT imaging) or (fluorescence-guided NEXT surger*) or (intraoperative* NEAR/3 fluoresc*) or (intra-operative* NEAR/3 fluoresc*)):ti,ab,kw | 514 |
| 10 | #1 or #2 or #3 or #4 or #5 or #6 or #7 or #8 or #9 | 5239 |
| 11 | MeSH descriptor: [Head and Neck Neoplasms] this term only | 2855 |
| 12 | MeSH descriptor: [Squamous Cell Carcinoma of Head and Neck] this term only | 463 |
| 13 | MeSH descriptor: [Otorhinolaryngologic Neoplasms] this term only | 50 |
| 14 | MeSH descriptor: [Laryngeal Neoplasms] this term only | 391 |
| 15 | MeSH descriptor: [Pharyngeal Neoplasms] explode all trees | 1143 |
| 16 | MeSH descriptor: [Neoplasms] explode all trees | 112562 |
| 17 | MeSH descriptor: [Mouth Mucosa] this term only | 901 |
| 18 | MeSH descriptor: [Pharynx] explode all trees | 1674 |
| 19 | MeSH descriptor: [Larynx] explode all trees | 1012 |
| 20 | MeSH descriptor: [Otolaryngology] this term only | 111 |
| 21 | (otorhinolaryngolog* or otolaryngolog* or head or heads or neck* or tonsil* or throat* or (nasal NEXT cavit*) or laryn* or glotti* or (vocal NEXT cord*) or pharyn*):ti,ab,kw | 77212 |
| 22 | #17 or #18 or #19 or #20 or #21 | 78599 |
| 23 | #16 and #22 | 5909 |
| 24 | ((otorhinolaryngolog* or otolaryngolog* or head or heads or neck* or tonsil* or throat* or (nasal NEXT cavit*) or laryn* or glotti* or (vocal NEXT cord*) or pharyn*) NEAR/3 (cancer* or tumo?r* or neoplasm* or carcinoma* or malignan*)):ti,ab,kw | 10322 |
| 25 | (HNSCC or SCCHN):ti,ab,kw | 1470 |
| 26 | #11 or #12 or #13 or #14 or #15 or #23 or #24 or #25 | 12872 |
| 27 | #10 and #26 | 74 |

**ClinicalTrials.gov**

**Condition or disease:**

((otorhinolaryngology OR otorhinolaryngologic OR otolaryngology OR otolaryngologic OR head OR neck OR tonsil OR throat OR nasal cavity OR larynx OR laryngeal OR pharynx OR pharyngeal) AND (cancer OR cancers OR tumor OR tumors OR tumour OR tumours OR neoplasm OR neoplasms OR carcinoma OR carcinomas)) OR HNSCC OR SCCHN

**Intervention/Treatment:**

colouring OR coloring OR fluorescent dye OR photosensitising OR photosensitizing OR fluorescent marker OR fluorescent markers OR aminolevulinic acid OR 5-aminolevulinic acid OR 5-ALA OR indocyanine green OR ICG OR optical imaging OR fluorescence imaging OR fluorescence-guided surgery OR intraoperative fluorescence

**WHO ICTRP**

**Condition**

((otorhinolaryngology OR otorhinolaryngologic OR otolaryngology OR otolaryngologic OR head OR neck OR tonsil OR throat OR nasal cavity OR larynx OR laryngeal OR pharynx OR pharyngeal) AND (cancer OR cancers OR tumor OR tumors OR tumour OR tumours OR neoplasm OR neoplasms OR carcinoma OR carcinomas)) OR HNSCC OR SCCHN

**Intervention**

colouring OR coloring OR fluorescent dye OR photosensitising OR photosensitizing OR fluorescent marker OR fluorescent markers OR aminolevulinic acid OR 5-aminolevulinic acid OR 5-ALA OR indocyanine green OR ICG OR optical imaging OR fluorescence imaging OR fluorescence-guided surgery OR intraoperative fluorescence
